# Three-Dimensional Printed Piezoelectric Array for Improving Acoustic Field and Spatial Resolution in Medical Ultrasonic Imaging

**DOI:** 10.3390/mi10030170

**Published:** 2019-02-28

**Authors:** Zeyu Chen, Xuejun Qian, Xuan Song, Qiangguo Jiang, Rongji Huang, Yang Yang, Runze Li, Kirk Shung, Yong Chen, Qifa Zhou

**Affiliations:** 1 College of Mechanical & Electrical Engineering, Central South University, Changsha 410083, China; zeyuchen@usc.edu; 2 Department of Biomedical Engineering, University of Southern California, Los Angeles, CA 90089, USA; xuejunqi@usc.edu (X.Q.); runzeli@usc.edu (R.L.); kkshung@usc.edu (K.S.); 3 Department of Mechanical and Industrial Engineering, The University of Iowa, Iowa City, IA 52242, USA; xuan-song@uiowa.edu (X.S.); 4 School of Electro-Mechanical Engineering, Guangdong University of Technology, Guangzhou 510006, China; qiangguojiang@gdut.edu.cn (Q.J.); rongji.huang@hotmail.com (R.H.); 5 Epstein Department of Industrial and Systems Engineering, University of Southern California, Los Angeles, CA 90089, USA; yang610@usc.edu (Y.Y.); 6 Roski Eye Institute, University of Southern California, Los Angeles, CA 90089, USA

**Keywords:** 3D Printing, piezoelectric array, ultrasonic transducer, ultrasonic imaging

## Abstract

Piezoelectric arrays are widely used in non-destructive detecting, medical imaging and therapy. However, limited by traditional manufacturing methods, the array’s element is usually designed in simple geometry such as a cube or rectangle, restricting potential applications of the array. This work demonstrates an annular piezoelectric array consisting of different concentric elements printed by Mask-Image-Projection-based Stereolithography (MIP-SL) technology. The printed array displays stable piezoelectric and dielectric properties. Compared to a traditional single element transducer, the ultrasonic transducer with printed array successfully modifies the acoustic beam and significantly improves spatial resolution.

## 1. Introduction

With exceptional piezoelectric, dielectric, and electronics properties, piezoelectric materials have been the focus of significant interest in the botch industry and academic fields. The wide applications ranging from signal sensor and energy harvesting devices to electromechanical actuator [1,2,3]. Among this material, piezoelectric ceramic with high piezoelectric constant and electromechanical coupling coefficient can effectively convert electrical signals into mechanical vibrations and vise versa, which results in obvious advantages in drug delivery, particle manipulation, ultrasonic imaging, and therapy [4,5,6,7]. The corresponding ceramic array with complex geometry has great potential to improve the performance of piezoelectric devices. For example, a piezoelectric array incorporating a hexagonal shape element demonstrated the evenly distributed sidelobe compared with rectangular shape element for nondestructive testing [8]. However, a piezoelectric array with complex geometry is challenging using traditional manufacturing methods such as dicing and etching [9,10]. In this regard, digital, additive, and automatic printing technologies offer a promising approach.

Additive manufacturing, or more commonly, 3D printing technology, is widely considered a revolutionary manufacturing technology. During past decades, direct inkjet based printing, extrusion-based direct write technique, and light exposure based Stereolithography Apparatus (SLA) have been used in ceramic component fabrication [11,12,13,14,15,16,17,18,19,20]. SLA involving the light exposure on photocurable polymer allows for the manufacture of complex geometry layer-by-layer with small resolution (X-Y resolution < 25 μm). It is compatible with multilaterals and composites printing, offering a distinct advantage for integrating functional materials in 3D objects [21,22,23]. Therefore, SLA has significant promise in a broad range of fields including mechanical and biomedical engineering. In previous work, Xuan et al. reported composite fabrication using Mask-Image-Projection-based stereolithograhpy integrated with tape-casting (MIP-SL) [24]. This method has been used for manufacturing a variety of materials such as resin and alumina ceramic. With MIP-SL method, we fabricated piezoelectric ceramic nanoparticles into 3D objects in previous work [25]. Since the decrease in surface energy due to a reduction in surface area is the main driving force for ceramic sintering, the nanoparticle-based fabrication results in improved piezoelectric property. After a specifically designed post processing, the 3D objects display the abilities on energy focusing and ultrasonic sensing. This method has great potential in applications of piezoelectric devices. The single element focused transducer concentrates an ultrasonic beam at a certain point (focus zone) with constant distance away from the transducer. The focus point has the highest intensity and the smallest lateral resolution. However, the lateral resolution at other positions besides except the focus point is unsatisfactory for ultrasonic imaging. Compared to a single element transducer, an array transducer has the potential to improve the focus zone and lateral resolution [26,27].

**Figure 1:**
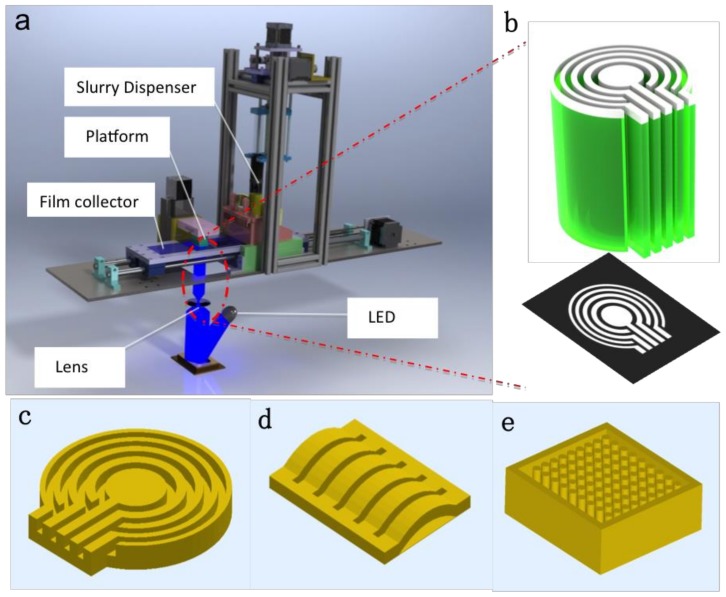
(**a**) sketch of Mask-Image-Projection-based Stereolithography system. (**b**) Green part controlled by image pattern. (**c**–**e**) 3D model designed by SolidWork.

In this study, we present here the design, fabrication, and post processing of piezoelectric array with piezoelectric effect and precisely controlled geometry using the MIP-SL system (Figure 1a). The 3D model was produced by Solidworks (Figure 1c,d,e). A Digital Micromirror Device (DMD) controlled the image pattern projected on the slurry (Figure 1b). An annular array with a limited number of elements was assembled in the ultrasonic array transducer that improved the lateral resolution and depth of field (focus zone). The resulting piezoelectric array structures are mechanically robust in the device, with stable dielectric and piezoelectric properties. Overall, the additive manufactured piezoelectric array enables improved performance and differing function of the corresponding device.

## 2. Materials and Methods

The BaTiO_3_ Nano powder (solid loading, 100 nm, Sigma-Aldrich St. Louis, MO, USA) was used as the raw materials. To modify the powder surface, an azeotropic mixture and dispersant (Triton x-100, Sigma-Aldrich, Saint Louis, MO, USA 0.5–0.8 wt.% on a dry weight basis of ceramic powders) were mixed with the powder by planetary mill (pulverisette 5, FRITSCH Idar-Oberstein, Germany) with 200 rpm rotation speed for 12 h. The azeotropic mixture consisted of methylethylketone (66 *v*/*v*%, 99%, MEK, Sigma-Aldrich, Saint Louis, MO, USA) and ethanol (34 *v*/*v*%, 99.5%, Sigma-Aldrich, Saint Louis, MO, USA). The mixture with powder was then dried at 50 °C for 12 h. After the evaporation of the solvent in the dispersion, deagglomerated BaTiO_3_ powders with dispersant adsorbed on to their surface can be obtained.

**Figure 2:**
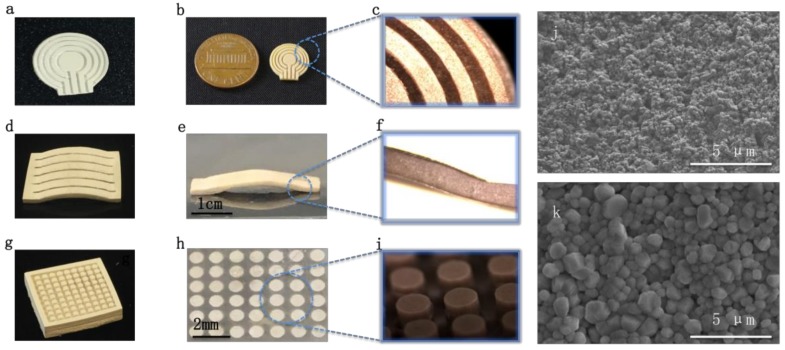
(**a**)(**d**)(**g**) Green-part fabricated by MIP-SL system. (**b**)(**e**)(**h**) Optical images of piezoelectric array after sintering. (**c**)(**f**)(**i**) Details of the array under microscope. (**j**) Scanning electron microscope image of printed sample after debinding process. (**k**) SEM image of printed sample after sintering process.

The deagglomerated BaTiO_3_ powder was mixed with a photocurable resin (SI500, EnvisionTec Inc., Ferndale, MI, USA) by ball milling for 1 h. The solid loading was 70 wt.%. MIP-SL system was used to fabricate 3D green part with the slurry. When the slurry was exposed under visible light, the photocurable resin in the slurry produced a cross-linked matrix, forming a strong bond between BaTiO_3_ powders and polymer network. The 3D model was produced by Solidworks (Figure 1a). A Digital Micromirror Device (DMD) controlled the images pattern projected on the slurry (Figure 1b). After the layer-by-layer process, a 3D green part was fabricated (Figure 2b).

The post-processing steps involved organic binder removal and high-temperature sintering. The 3D green part was debinded in a muffle furnace with Argon under 600 °C for 3 h. After the furnace cooling, the debinded part was put in a regular muffle furnace with air at 1300 °C for 2 h.

The sintered samples and bulk samples were poling under 30 kV/cm at 100 °C for 30 min. Dielectric constant and dielectric loss (tanδ) measured by an impedance analyzer (Agilent 4294A, Santa Clara, CA, USA). Density was measured by ASTM B962-14 standard. Piezoelectric constant was measured by d33 meter (APC International, Ltd., Mackeyville, PA, USA).

**Figure 3:**
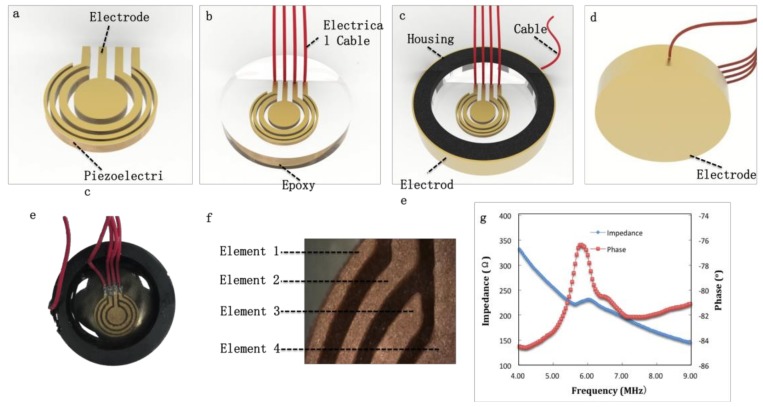
(**a**–**d**) Structure of annular array transducer. (**e**) Optical image of annular array transducer. (**f**) Four elements of annular array. (**g**) Element’s spectrum of impedance and phase.

The array transducer was fabricated using the annular array. Epoxy (Epo-Tek 301, Billerica, MA, USA) was filled into the kerf between each element. A 150 nm Cr/Au layer was sputtered on the annular array to serve as the electrode Figure 3a. Four electric cables were connected on the elements with conductive epoxy (E-Solder 3022, Von Roll Isola Inc., New Haven, CT, USA) shown in Figure 3b. The housing was an Aluminum Silicate. Epoxy (Epo-Tek 301) with an acoustic impedance of ~6 MRayl was inserted into the housing to serve as the backing layer (Fgiure 3c). A Cr/Au layer was sputtered on the other side of the annular array with a cable attached on the layer (Figure 3d.) After that, a 10μm-thick parylene was vapor-deposited by Specialty Coating System (SCS, Indianapolis, IN, USA) on the other Au surface to protect the transducer array. The impulse-echo response of each element in the array transducer was measured by an ultrasonic system consisting of PC, gage card, JSR500, function generator, motor and quartz target (Figure 5).

The transducers were driven by JSR500 (Ultrasonics, Imaginant Inc., Pittsford, NY, USA) and triggered by the function generator with a pulse repetition frequency (PRF) of 1kHz. The ultrasonic signals were filtered by an analog band-pass filter. A 12-bit digitizer card (ATS9360, Alazartech, Montreal, QC, Canada) with a 1.8 GHz sampling rate was used to record the signals (Figure 5). To obtain a 2D image, the transducers were mounted on a stepper motor (SGSP33-200, OptoSigma Corporation, Santa Ana, CA, USA) for mechanical scanning with 36 μm increment.

## 3. Results and Discussion

### 3.1. Characterization of Sintered-Parts

An annular array (Figure 2a,b,c), self-focused linear array (Figure 2d,e,f), and cylinder array (Figure 2g,h,i) were fabricated using MIP-SL and the post-processing method. Figure 2a,d,g show the green-part involving piezoelectric nanoparticles printed by the MIP-SL system. Figure 2b,e,h display the piezoelectric array after post-processing and Figure 2c,f,I are the optical images of the array under a microscope. The scanning electron microscope images of the debinded part and sintered sample were shown in Figure 2j and k, respectively. The figures show that after post-processing, the density of printed ceramics increased obviously. Limited by traditional machining technology, the piezoelectric elements of array are usually designed in square or rectangular shape with fixed kerf, while the three types of printed array demonstrates more flexibility of complex geometry. The specific designed annular array can not only focus ultrasound, but also improve the depth of acoustic field (Focus zone). The design and application will be discussed later.

A set of cylindrical samples with 10mm diameter and different thickness (400 μm, 800 μm, 1.2 mm, 1.6 mm) were fabricated using the same fabrication process. The bulk cylindrical samples and the printed array with complex geometry were used to characterize the printed piezoelectric ceramics. The poling filed was 30 kV/cm at 100°C for 30 min. The dielectric constant was 1300~13,500, the dielectric loss was 0.018~0.02. Density was 5.62~5.64 g/cm^3^. Piezoelectric constant was 146~160 pCN^−1^.

### 3.2. Annular Array Transducer

Here we designed an annular array and fabricated it with the MIP-SL method. A transducer using the printed annular array with a limited number of elements can provide an improved depth of field and improve lateral resolution over the field, when compared with a single element transducer with the same total aperture and focal length.

The annular array consists of 5 concentric elements and the outermost element can prevent damage during sample transfer. After sintering, the outermost element was removed from the annular array. The thickness of the array was 400 μm. The area and center frequency of 4 working elements were designed as 13.5 mm^2^ and 6 MHz.

An ultrasonic transducer was fabricated using the printed annular array (Shown in Figure 3). Figure 3f numbers each element after sintering, the actual area can be found in Table 1. Impedance analyzer (Agilent 4294A) was used to measure the spectrum of impedance and phase. The spectrum of element 1 is shown in Figure 3g. The other elements have the similar spectrum. The electromechanical coupling coefficient of piezoelectric materials is defined as the ratio of the mechanical energy accumulated in response to an electrical input or vice versa, which can be expressed in the following equation:

(1) $$k_{t}=\sqrt{\frac{\mathrm{Mechanical}\;\mathrm{energy}\;\mathrm{stroed}}{\mathrm{Electrical}\;\mathrm{energy}\;\mathrm{applied}}}$$

(2) $$=\sqrt{\frac{\mathrm{Electrical}\;\mathrm{energy}\;\mathrm{stored}}{\mathrm{Mechanical}\;\mathrm{energy}\;\mathrm{applied}}}$$

k_t_ can be formulated as [28]:

(3) $$k_{t}=\sqrt{\frac{{\pi f}_{r}}{2f_{a}}\times \cot\frac{{\pi f}_{r}}{2f_{a}}}$$

where $f_{r}$ is resonant frequency, and $f_{a}$ is anti-resonant frequency. For example, Figure 3g shows the spectrum of the element in the array transducer. The $f_{r}$ and $f_{a}$ are 5.54 MHz and 6.1 MHz, respectively, and the corresponding k_t_ is 46.5%. The coupling coefficient of printed bulk ceramics and other array elements were measured with the same method and the values do not have an obvious difference (46.3~46.5%). The results demonstrate that both the printed bulk ceramics and array have stable dielectric and piezoelectric properties, which do not change obviously in their geometry and thickness.

**Figure 4:**
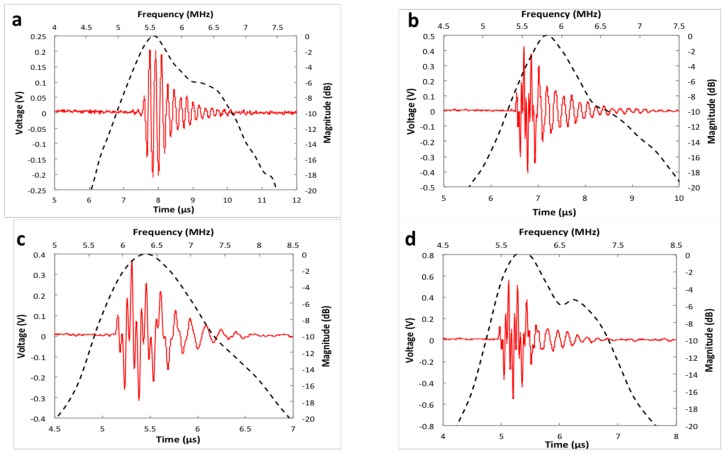
Pulse-echo waveform (solid line) and normalized spectrum of element 1 (**a**), element 2 (**b**), element 3 (**c**) and element 4 (**d**).

Each element can transfer electrical impulses to mechanical oscillation and then generates ultrasound. After a target reflects the ultrasound, the elements converted the returned echoes back into electrical impulses, which could be further processed to from an ultrasonic image. The impulse-echo response was measured by an ultrasonic system. Figure 4 represents the pulse-echo waveform (solid line) in time domain and normalized spectrum in frequency domain of each element. Table 1 shows the measured pulse and echo characteristics for all array elements. V_PP_ is peak-to-peak voltage. The variations between four elements may be caused by the debinding and sintering process, which lead to inaccuracy of the geometry.

**Figure 5:**
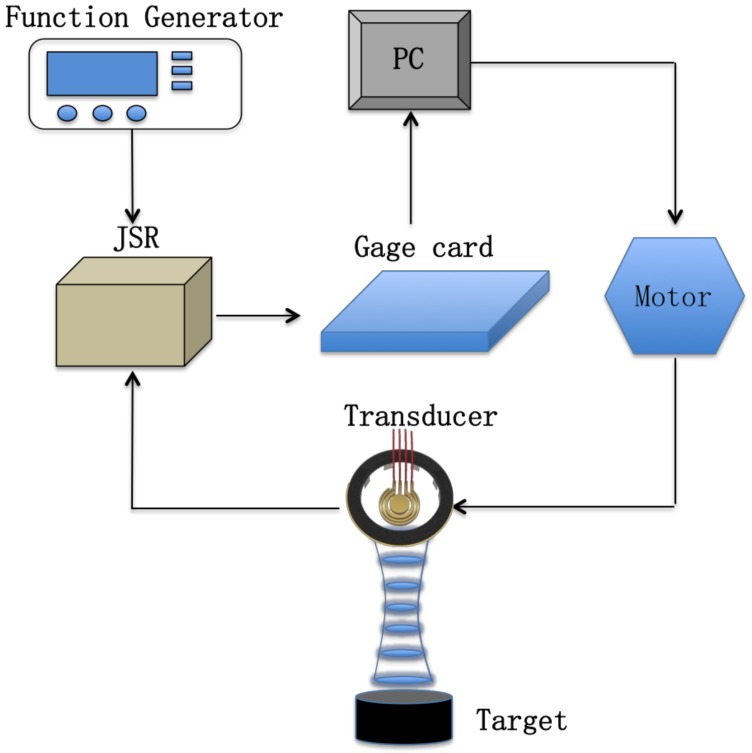
Schematic of the test system setup for pulse-echo detecting and ultrasonic imaging.

**Figure 6:**
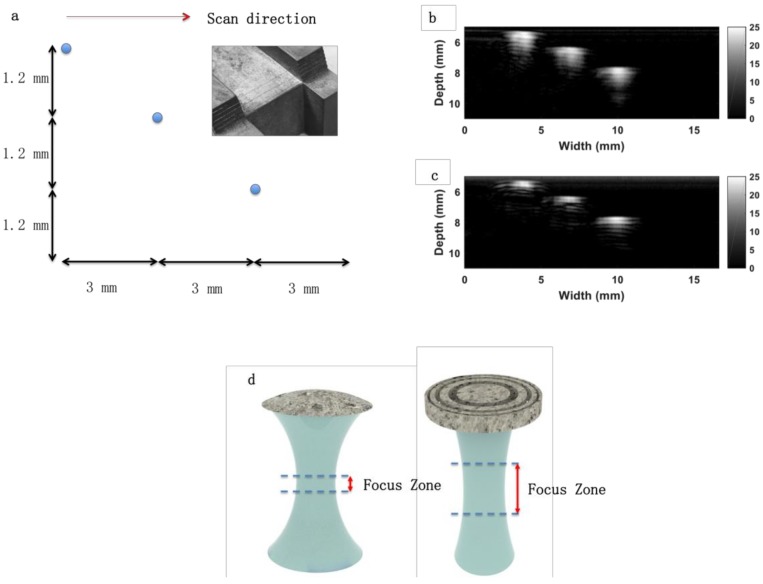
(**a**) Sketch of the wire phantom. The inset is the optical photo of the wires. (**b**) Phantom imaging by single element transducer. (**c**) Phantom imaging by annular array transducer. (**d**) Schematic of the acoustic beam for single element (**left**) and annular array (**right**).

To test the performance of the annular array transducer, the quartz target in Figure 5 is replaced by a wire phantom with 3 tungsten wires (50 μm in diameter). Figure 6a displays the sketch of the wire phantom. The phantom was imaged by the annular array transducer and a single element transducer, respectively, to assess the lateral resolutions. The single element has the same aperture (diameter) with the array transducer and 6MHz center frequency.

**Figure 7:**
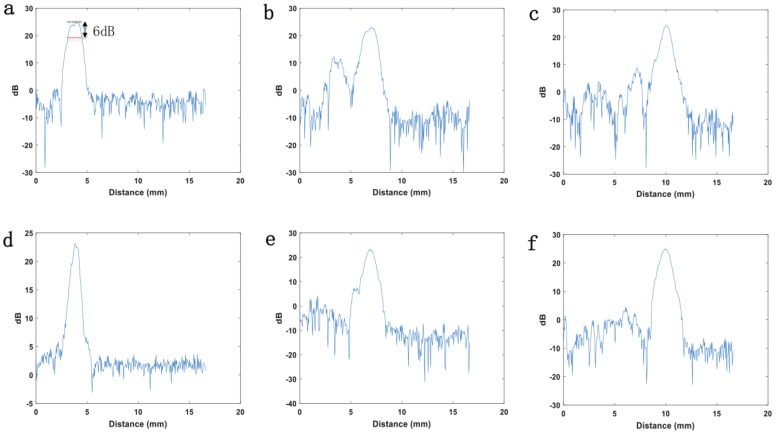
Signal magnitude (dB) versus scanning distance for lateral resolution measurement. The resolution was measured when the depth between wire and single element were 5.6 mm (**a**), 6.8 mm (**b**) and 8 mm (**c**). The resolution was then measured when the depth between wire and annular array were 5.6 mm (**d**), 6.8 mm (**e**) and 8 mm (**f**).

The array transducer is controlled by a stepper motor. The scan direction is labeled as a red arrow shown in Figure 6a. As the transducer changes scanning distance, it detects the wire at different depths. After one element transmits an initial signal, an element receives the echo signal. This process is a transmit-to-receive combination. So in this study, 4 elements have 16 transmit-to-receive combinations (4 × 4 = 16). These transmit-to-receive combinations were processed with a specific beam forming technology reported in reference [29] for the ultrasonic images. Figure 6b,c demonstrates the phantom images of the single element and annular array transducer when the dynamic range was −25dB. The phantom image of the array transducer (Figure 6c) displays an improved signal to noise ratio compared to the single element transducer (Figure 6b). Figure 7 shows the dB (signal magnitude) versus scanning distance for single element (Figure 7a,b,c) and annular array (Figure 7d,e,f) transducer when the wires were set at different distances away from the transducer. As Figure 7a shows, the lateral resolution is the intercept (read dash line) when dB (magnitude) is the maximum value (black dash line) minus 6 dB. Table 2 shows the lateral resolutions at a different depth of different transducers. Because the ultrasonic beam diverges quickly when the depth is larger than 8.0 mm, we studied the lateral resolution within 8 mm.

The results indicate that the focus point of the single element transducer is located at 8 mm away from the transducer, when the lateral resolution is 1.1 mm. But the lateral resolution (beam width) of the single element at another depth is obviously larger than 1.1 mm. For example, the lateral resolution at 5.6 mm depth is 1.4 mm. In contrast, the array transducer provides similar and small lateral resolutions (beam width) less than 1.1 mm from 5.6 mm to 8 mm. A sketch of ultrasonic beams generated by two different transducers is shown in Figure 6d. With the tunable focus zone, medical imaging for the target at a different depth can be achieved. Not only medical imaging, but also particle manipulation and ultrasonic therapy can benefit from the boarder focus zone. There are two downsides to this method. Firstly, the density of printed samples is lower than bulk materials. Secondly, the debinding and sintering process would lead to defects and inaccuracy of the geometry.

## 4. Conclusions

Using Mask-Image-Projection-based Stereolithography, photocurable resin and nano ceramic particles can be 3D-printed into arbitrarily shaped arrays. After post-processing, the dense ceramic arrays display stable piezoelectric and dielectric properties. A specifically designed annular array was printed with our method. Each element of this array can convert electric signals to mechanical vibration, and vice versa. With a beam forming, the ultrasonic transducer with printed array improved the acoustic field and signal-to-noise ratio, which resulted in a longer focus zone and the smaller lateral resolution. This array transducer with tunable focus zone and resolution has many benefits in medical imaging, non-destructive detecting and high intensity focused ultrasound. The 3D printing enabled morphology of the piezoelectric array can lend itself to a range of potential applications in the medical transducer, composite design, and wearable and implantable electronics.

## Figures and Tables

**Table 1:** The measured pulse and echo characteristics for all elements.

Characteristics	Element 1	Element 2	Element 3	Element 4
Center Frequency (MHz)	5.72	5.86	6.39	6.12
−6 dB Bandwidth (%)	19.6	12.9	19.8	23.6
V_pp_ (mV)	402	793	626	1039
−20 dB Pulse Length (ns)	1940	1621	989	2949
Area (mm^2^)	13.7	13.2	13.6	13.5

**Table 2:** Resolution of single element and annular array.

Depth (mm)	Resolution (mm)	
	Single Element	Annular Array
5.6	1.4	1
6.8	1.5	1.05
8	1.1	1.1
